# The Institutional Library

**Published:** 1920-10-02

**Authors:** 


### THE INSTITUTIONAL LIBRARY.

**Gonococcal Infection in the Male.** By Norman Lumb, O.B.E., M.B., B.S. Pp. 328. (John Bale, Sons and Danielsson, Ltd. Price 25s. net.)

Mr. Lumb gives a complete record of the best knowledge that up to the present we possess. From the development of the male organs of generation down to the destruction of their structure and purpose there is nothing lacking in the hideous story of an uncured case of the disease. Its potentiality for evil is fully recognised, and the urgent need of prevention and cure dwelt upon with emphasis. As a relief to this dark picture comes the description, admirable both in letterpress and illustration, of the means and results placed within our reach by advancing knowledge. The urethroscope rightly occupies a prominent position in the book, and its necessity in the adequate treatment of gonorrhœa would serve to point the moral that all cases should at present be dealt with from start to finish by the specialist.

With regard to detoxicated vaccines, the author is favourably impressed by the results obtained, but he is of opinion that whatever permanent value may ultimately be attained by this method of treatment, we do not up to the present possess in them a reliable source of cure. Chemo-therapeutic treatment is in its infancy, its schemes of treatment are complex, and a final verdict cannot at present be given. Electro-chemical treatment also is in the experimental stage. In the routine treatment by irrigation with potassium permanganate Mr. Lumb is altogether in favour of washing through into the bladder in cases of anterior urethritis, and is in accord with most authorities in denying that risk is run of infecting the posterior urethra. No one concerned in the treatment of gonorrhœa can afford to ignore the book.

**Common Infections of the Kidneys.** By Frank Kidd, F.R.C.S. Oxford Medical Publications: Frowde, and Hodder and Stoughton. 1920. Pp. 331. Price 18s. net.

When the present reviewer was a student the teaching about bacterial infections of the kidney was very scanty and unsatisfactory: and he is by no means sure that even now a proper amount of attention is paid in the medical schools to these very common disorders. The introduction of the cystoscope—which British surgeons were extremely slow and backward in adopting—and the progress of bacteriology have thrown floods of light on these conditions; but it is still only too true that few surgeons, outside the specialists in urinary diseases, have a really sound knowledge of them. A monograph devoted to these important kinds of infection (Mr. Kidd excludes tuberculosis) is, therefore, very welcome, and doubly so from the extensive clinical material which the author has had at his disposal and the excellent use to which he has put it. Mr. Kidd thinks for himself, and will be bound by no hoary shibboleth if it conflicts with his experience. This is a great merit, though it leads him at times into the enunciation of dogmata which some day he may have to recant; he holds his opinions very positively and is rather impatient of, even rude to, colleagues who think differently. Particularly interesting are his hypothesis as to the origin of hydrocele; and his suggestion that the variety of tuberculous epididymitis with acute swelling of the testicle, described in the text-books, is in reality a *B. coli* infection and not tuberculous at all. This view is new to the writer, and on looking back over some past cases he is inclined to think there is much to be said in favour of it. Altogether this book should appeal strongly to the practising man, whether surgeon or general practitioner, for it is full of clinical wisdom set forth with acumen. As usual, the publishers have done their full share in making it attractive.

**Cunningham's Manual of Practical Anatomy.** Revised and edited by Professor Arthur Robinson. Seventh edition. Vol. III., "Head and Neck." (London: Henry Frowde, Hodder and Stoughton. Price 12s. 6d. net.)

Owing to considerable additions to the text and to the number of illustrations this manual has expanded to three volumes. Previous editions have been remarkable for the wealth and excellence of their illustrations; and the addition of more diagrams and radiographs has now made it a work of superlative value to the student of regional anatomy. This edition cannot fail to maintain the popularity which Cunningham's manual has experienced in the past. The instructions for dissection have been printed in a distinctive type, and to a great extent the book serves the double purpose of dissecting-room hand-book and text-book of anatomy.

